# 

**Published:** 1804-07-01

**Authors:** 


### THE MEDICAL AND PHYSICAL JOURNAL.

THE

### MEDICAL

AND

### PHYSICAL

### JOURNAL.

CONDUCTED BY

T. BRADLEY, M.D.

R. BATTY, M.D.

AND

A. A. NOEHDEN, M.D.

VOL. XII.

FROM JUNE TO DECEMBER, 1804.

5118.

*LONDON:*

PRINTED FOR RICHARD PHILLIPS, NO. 71, ST. PAUL'S CHURCH YARD.

[ By William Thorne, Red Lion Court, Fleet Street. ]

W
1
ME 182

[ Entered at Stationers Hall. ]

### To CORRESPONDENTS.

Mr. Chevalier's Treatise on Gun-Shot Wounds will be noticed in our next Number.

A Communication, with a Drawing, has been received from Mr. Edgar.

